# Discovering maritime-piracy hotspots: a study based on AHP and spatio-temporal analysis

**DOI:** 10.1007/s10479-023-05352-z

**Published:** 2023-04-28

**Authors:** Marios Tsioufis, Antonios Fytopoulos, Dimitra Kalaitzi, Thomas A. Alexopoulos

**Affiliations:** 1 Department of Economics, School of Economics and Technology, University of Peloponnese, Tripolis Campus, Tripoli, Greece; 2 Department of Chemical Engineering, KU Leuven, Celestijnenlaan 200F, 3001 Leuven, Belgium; 3 School of Chemical Engineering, NTUA, Athens, Greece; 4 Aston University Engineering Systems and Management: Aston University School of Engineering and Technology, Birmingham, West Midlands United Kingdom

**Keywords:** Maritime piracy, AHP, Spatio-temporal data analysis, Time-varying granger causality, Oil tankers

## Abstract

Shipping is the backbone of international trade and oil companies want their oil tankers to arrive safely. The safety and security of international shipping of crucial elements such as oil has always been important aspect in the landscape of piracy. The implications of piracy attacks are linked with loss of cargo or personnel, economic and environmental catastrophe. Despite maritime piracy being a major concern to international trade, no comprehensive study considers the causing factors and spatio-temporal patterns that influence the choice of attack areas. Thus, this research expands our understanding on the areas where piracy mainly occurs, and its underlying causes. To achieve these objectives, AHP and spatio-temporal analysis applied by using data from the National Geospatial-Intelligence Agency. The results indicate that territorial waters are preferable areas; thus, pirates prefer to attack the ships close to the coastline fewer times near ports, and rarely in international waters. This is in line with the spatio-temporal analysis results that show that pirates except for the Arabian sea prefer to hit close to the coastline of countries that face political instability, lack of properly functioning government and extreme poverty. Moreover, pirates in certain areas are influenced by the activity and the information from other pirates, which can be used as tool from the authorities e.g., derive information from pirates that have been arrested. Overall, this study contributes on the literature of maritime piracy, and it could be used to enhance security and build tailored defense strategies in perilous water areas.

## Introduction

Global transportation, particularly sea transportation of crude oil, plays an important role in global oil industry supply chain management. However, maritime transportation can be dangerous e.g., piracy, natural disasters, vessel collisions (Lim et al., 2018). Maritime piracy is a pressing global issue and can be defined as “an act of boarding or attempting to board any ship with the apparent intent to commit theft or any other crime and with the apparent intent or capability to use force in the furtherance of that act” (International Maritime Bureau, 2009, cited in Hassan & Hasan, 2017, pp. 4–5). Despite that the number of worldwide maritime piracy incidents per year declined, it continues to be a threat impacting significantly companies dealing with the production and distribution of oil, leading to several social and political impacts, since oil and all energy, either primary or secondary, sources (e.g. natural gas, renewables, electricity and so on), are important production factors, they are interconnected within our society, and have strong environmental externalities (Agliardi et al., 2019; Alexopoulos, 2017).

195 maritime piracy attacks reported in 2021, and the Gulf of Guinea has been indicated as the world’s most dangerous shipping route (IMB, 2021). One of the most important causes of piracy in this area is the opportunity i.e., favorable geophysical attributes and laws against maritime piracy (Murphy, 2007). For example, the high unemployment rates, inadequate law enforcement, unregulated oil market capacity, corruptible officials, enable pirates in the Gulf of Guinea to move stolen products back onto legitimate markets (European Union, 2020). This area is a transit point in the illicit trade of oil out of Africa that costs annually African countries around $524 million on counter-piracy efforts (Stable Seas, 2021). COVID-19 increased sea-piracy incidents in Nigeria (Gold et al., 2023). Similarly, the Southern Gulf of Mexico has recently received attention as four attacks reported in a span of 11 days in April 2020. The maritime piracy in the Gulf of Mexico and Caribbean can be attributed to a number of factors such as the Covid-19 pandemic, lack of the necessary resources and capabilities to adequately detect and respond to these attacks (Drake, 2021). It is supported that this region will continue to be a hotspot in the future, but more will also emerge. For example, Russia's war in Ukraine that caused the rising of crude oil prices might lead to piracy attacks on tankers in Asia (Concepcion, 2021). All these incidents lead to severe risks in terms of safety, delays and increasing cost (e.g. the insurance cost of oil tankers is rising, cost of the wide range of interventions, such as naval patrols cost of re-routing); currently worldwide is a loss of nearly $25 billion per year (Fan et al., 2022).

Despite the importance of maritime piracy and the rise of the incidents in 2020, it is worth noting that there is not much research in the specific area and there are a few outdated studies concerned with patterns of maritime attacks (Marchione & Johnson, 2013; Townsley & Oliveira, 2015). Moreover, most of the papers focus only on a particular area to trace the evolution of maritime piracy e.g., Nigeria (Otto, 2014) and lacked a holistic view of maritime piracy. Last but not least, despite a few studies identifying the maritime piracy prone areas, but they do not explore the characteristics of the piracy within these vulnerable areas. Therefore, there is a lack of global-based studies that provide a complete overview of maritime incidents, in terms of the hotspot areas, and the characteristics of incidents within the identified areas. In response to this research gap, the aim of this paper is to analyze maritime piracy concentration and hot spot dynamics to better inform piracy prevention and reduction strategies. In this sense, our paper is guided by the following research questions (RQs):

- *RQ1* Where are the hotspots of maritime piracy?
- *RQ2* What are the main causes of maritime piracy?
- *RQ3* Are there any patterns and dependencies in the timing and location of incidents of maritime piracy?

Therefore, this study aims to explore maritime piracy (attempted and completed attacks) with a particular focus on oil tankers rather than vessels carrying other products. This type of commodity is desirable for pirates as it has high value and they can easily sell it on the black market and the ship itself is also valuable (Robitaille et al., 2020).

A hybrid two-phased method applied to answer the above RQs of the study. First, an Analytic hierarchy process (AHP) used in this paper, developed by Saaty in 1977 which is an effective decision-making tool used in complex situations (Pereira & Bamel, 2022), in order to set priorities by developing a weighted multi-criteria model to identify the prominent factors of maritime piracy. This model contains all the influencing factors (i.e., zone, security, time, distance) according to their importance (weighted factor approach).

Moreover, kernel density estimation (KDE) is applied to estimate the concentration of maritime piracy incidents at each grid and visualize it, attempting to identify potential regional patterns. Apart from spatial hotspot identification, in this study the temporal component of piracy events is considered in order to explore whether dynamic causal relationships exist. Last but not least, a joint spatial and temporal analysis is introduced to shed light to underlying common pirate networks or persons.

The recognition of ‘hotspots’ in terms of time and space concentrations will enable the deployment of resources to prevent the generation of piracy attacks. Moreover, the identification and explanation of emerging piracy patterns enable the effective planning of a functional environment in order to minimize the vulnerability of certain locations to maritime piracy. The paper begins by examining the existing literature and methodologies applied to explore maritime piracy. Then Sect. 3 discusses the methods employed by the present study to address the above RQs. Next, the results are presented and discussed in Sects. 4 and 5. The paper concludes with Sect. 7 that entails the conclusions, limitations of the study and opportunities for further research.

## Literature review

Between 2015 and 2020 most piracy attacks emerged in bulk carriers in March, April, and May in 2015 in Southeast Asian waters (Ece & Kurt, 2021). A recent study also highlighted that pirate attacks on ships are more prone to Southeast Asian and African waters (Nwokedi et al., 2022). Maritime piracy impacts the whole supply chain, and several companies demand assurance of security by identifying the factors that make these ships vulnerable to piracy.

The causes, implications and mitigations strategies of maritime piracy are discussed in the remainder of this section as well as the main methodologies and tools applied in this research field.

### Causes of maritime piracy attacks

There are various root causes of piracy which makes it a multidimensional phenomenon, and each cause are not stand-alone but are interconnected with one another. There are macro-level determinants (e.g. political, economic stability, socioeconomic conditions, geographic location, moral values) and micro-level determinants (e.g., vessel type, size, and voyage) of marine piracy (Jin et al., 2021). For example, Jiang and Lu (2020) developed a probability prediction model based on a Bayesian Network (BN) to investigate the causal factors of piracy in Southeast Asia and found that the main factors are the environment, ship's features and anti-piracy measures. Özdemir and Güneroğlu (2017) applied fuzzy AHP and fuzzy TOPSIS methods and identified the major causing factor of piracy namely economic insufficiency and as less significant factor the geographic location of the canals and straits.

### Maritime piracy implications

Several studies also focused on the implications of maritime piracy. For example, Fu et al. (2010) developed a simulation model to explain the financial implications (e.g., increased cost of insurance, increased costs associated with ships being forced to take alternate routes) on the global shipping industry. Some studies that particularly concentrated on Somali piracy found that piracy led to increased shipping cost and decreased trade volumes (Besley et al., 2015; Burlando et al., 2015). Similar outcomes observed by the study of Bensassi (2012) who supported that hijacking is linked with decrease in exports and Shepard and Pratson (2020) who found that higher pirate attack rate is associated with a 7.5-vessel reduction in tanker traffic. Other studies highlighted that piracy prone waters led decision makers to increase the voyage length in order to avoid high-piracy areas (Dinwoodie et al., 2013; Vespe et al., 2008). Last but not least, a few studies also highlight the impacts on seafarer’s wellbeing and the associated human cost (Abila & Tang, 2014; Seyle et al., 2018).

### Strategies to counteract maritime piracy

Apart from recognizing the oil theft causing factors and its impacts, studies also discussed some of the countermeasures. Romsom (2022) discussed the need for innovative technology, processes, and transnational collaboration that promote and align economic and policy regimes which is in line with other studies (e.g., Osinowo, 2015). Bouejal et al. (2014) implemented a Bayesian network that considers different characteristics e.g., potential target, environmental constraints to identify appropriate countermeasures. Another study applied a Fuzzy AHP and found that international convention and policy, defense strategies when sailing, hardware and software, security plan, response ability, and communication facilities are the main elements that need to be considered to minimize piracy attacks (Tseng et al., 2021). Studies also suggested that anti-piracy operations, regulations and policies as well as coordination at domestic, regional, and international level enable the minimization and control of piratical activities (Ahmad, 2020). The presence of naval bases is crucial in supporting maritime forces combat piracy (Danzell et al., 2023). There is a need to reconsider and improve the legal system for maritime piracy by strengthening the universal jurisdiction (Jin & Techera, 2021). Last but not least, Hasan and Hasan (2017) focused on the Gulf of Guinea and supported the need of exploration of piracy-prone zones, the knowledge of the particular ship types, the economic support particularly of youthful population and information-sharing are some of the strategies that can be utilized to combat piracy.

### Research approach and methodologies used to explore spatial and temporal patterns of maritime pirate attacks

Mathematical and agent-based models have been used to explore spatial and temporal patterns of maritime pirate attacks. Jakob et al. (2010) proposed a spatially explicit agent-based to help the visualization, anticipation and prevention of preventing pirate attacks. Vos Fellman et al. (2015) applied the social network analysis and visualized the maritime attacks by region, and its characteristics. Townsley and Oliveira (2015) utilized the rational choice theory and optimal foraging theory to explore space–time patterns of maritime piracy in Africa and confirmed that pirate activity clusters in space and time. Marchione et al. (2014) developed an agent-based model by focusing on the Gulf of Aden to investigate the geometry of shipping routes, seasonal variation of attacks, and show if the attacks differ by vessel type and state of registration, an approach that followed also by Mejia et al. (2009) who applied econometric analysis.

Tešić (2020) proposed a qualitative approach of computation-with-words approach to track maritime objects and identify attacks and decide on the countermeasures. Jin et al. (2021) classified piracy risk into macro-level and micro-level analyses and employed binary logistic regression model and found that small vessels and open registry vessels are more prone to be targeted by pirates. Desai and Shambaugh (2021) identified a pattern between the fish volumes caught using destructive and high-bycatch methods and piracy by conducting a geographically disaggregated analysis. Ofosu-Boateng (2017) focused particularly on oil maritime piracy. This study developed three models (Ordinal Logistic Regression, Bayesian Network Predictor, and Series Hazard Models) to forecast piracy attacks the next fifteen years in the Gulf of Guinea. Balogun (2018) applied the spectrum-based theory of enterprise, and Porter’s value chain to discuss oil theft, illicit business, and petro-piracy in the Gulf of Guinea.

Most of the studies focus on specific geographical areas (e.g., de Montclos, 2012; Nwalozie, 2020) thus they do not take a holistic approach for identifying global hotspots. Moreover, there are a few studies that rigorously explore in a quantitative way the causes of maritime piracy (Jiang & Lu, 2020; Özdemir & Güneroğlu, 2017). Previously published studies on the subject have been mostly restricted to show the patterns and the existence concentrations of maritime piracy without investigating in detail the determinants of their presence. In this sense, to the best of our knowledge, this study is unique as it explores the maritime piracy concentration by investigating clusters and causes of marine -piracy.

## Data and empirical methodologies

### Data description

In this study, AHP approach and spatial, temporal, and spatiotemporal analyses are performed, and described in the sub-sections below. This study used the National Geospatial-Intelligence Agency (NGIE) database that registers all the hostile acts worldwide against ships and mariners (NGIE, 2022). The focus of this study was only on attacks against fossil fuel transportation i.e., oil, (liquefied natural gas) LNG, oil products, etc., from 2000 until 2022. In total, 2145 relevant observations are found in the database. Each entry entails: the date, the location (coordinates and naval area), the attacker, the victim, and a description that shows if the hostile act was successful or not and if it took place underway or in position. The methods chosen in this study need the daily or monthly frequency of the incidents. However, the illicit oil flows are not officially recorded in that level of detail, so the current data are not appropriate for systematic analysis. To overcome this challenge, a proxy variable was selected i.e., a variable based on all the hostile acts against tankers because they disrupt directly or implicitly the supply of legitimate oil transportations in favor of illicit oil flows (Hatipoglu et al., 2022). In most cases, delaying fossil fuel transportation was the mildest repercussion, reaching total oil loss in the worst-case scenario.

### AHP methodology

AHP is a widely used multi-criteria analysis method that considers both quantitative and qualitative criteria and helps users make better decisions (Saaty, 1987). This method has been applied in various fields e.g., decision theory, conflict resolution, and neuroscience (Vargas, 1990). The method is a framework in which a problem can be solved based on three principles: decomposition, comparative judgments, and composition. AHP uses the Eigenvalue approach to the pairwise comparisons and allows the user to develop a numeric scale in order to measure the qualitative or quantitative performance of criteria (Vaidya & Kumar, 2006). Table 1 shows the fundamental scale developed by Saaty. This scale explains the way that the criteria will be ranked from 1 to 9 and their interpretation. It should be underlined, that the ranking of the criteria is at the user's discretion, according to the general objective (focus of the problem).

**Table 1:** The fundamental scale of relevant importance among criteria (Saaty, 1987)

Intensity of importance on an absolute scale	Definition	Explanation
1	Equal importance	The activities contribute equally to the objective described from the author
3	Moderate importance of one over another	Experience and judgment favor one activity over another
5	Essential or strong importance	Experience and judgment strongly favor one activity over another
7	Very strong importance	An activity is strongly favored, and it is dominance demonstrated in practice
9	Extreme importance	The evidence favoring one activity over another is of the highest possible order of affirmation
2, 4, 6, 8	Intermediate values between the two adjacent judgments	When compromise is needed
Reciprocals	If activity i has one of the above numbers Assigned to It when compared with activity j, then j has reciprocal value when compared with i	

AHP applied in this study to predict the behavior of the pirates/smugglers, which attack the ship, or the tankers carrying oil and then transport those ships to the endpoint. The endpoint i.e., final destination would be a warehouse or a safe port, in which the smugglers can offload the stolen oil and make the transaction with the buyer. The focus of this research is to identify the areas where pirates perform most of their attacks in order to help prosecution authorities enhance the degree of security in these specific regions. Based on previous studies four are the main factors that pirates consider before attacking a ship: (1) Security (i.e., if there are effective security measures for the smugglers or not—existence of prosecuting authorities), (2) Time (i.e., the time that the smugglers need for the operations), (3) Topography/area (e.g., wind, weather, underwater obstacles etc.), and (4) Fuel consumption.

Table 2 shows these criteria with respect to the overall focus of this study based on the numerical scale presented in Tables 1, 3, 4, 5 and 6 show the weights for alternatives, based on each criterion. For example, the topography criterion is considered to be “essential or strong importance” compared to time criterion.

**Table 2:** Pairwise comparisons of the criteria

Focus criterion	Security	Time	Topography	Fuel consumption
Security	1	8	4	7
Time	1/8	1	1/5	1/2
Topography	1/4	5	1	1
Fuel consumption	1/7	2	1	1

**Table 3:** Pairwise comparisons of alternatives with respect to Security criterion

Security	Ports	Territorial	International
Ports	1	1/7	1/3
Territorial	7	1	5
International	3	1/5	1

**Table 4:** Pairwise comparisons of alternatives with respect to Time criterion

Time	Ports	Territorial	International
Ports	1	4	9
Territorial	1/4	1	3
International	1/9	1/3	1

**Table 5:** Pairwise comparisons of alternatives with respect to Topography criterion

Topography	Ports	Territorial	International
Ports	1	3	8
Territorial	1/3	1	5
International	1/8	1/5	1

**Table 6:** Pairwise comparisons of alternatives with respect to Fuel Consumption criterion

Fuel consumption	Ports	Territorial	International
Ports	1	3	9
Territorial	1/3	1	3
International	1/9	1/3	1

Apart from the aforementioned causes of piracy, it has been supported that in terms of topography there are three areas that pirates/smugglers may prefer to perform their attack. These are: (1) Port areas, congested channels and checkpoints that the ship will pass through. (2) Territorial waters up to 12 nautical miles from the coastal baseline (without the port), (3) International waters which are located more than 12 nautical miles away from the costal baseline (Hastings, 2009). Based on these four criteria, underlying causes of piracy and the application of the AHP method, this study will be able to identify the area achieving the highest score which is the most preferable by pirates/smugglers. The procedure of setting weights in our work, is the one followed by Saaty [ref]. Additionally, the authors mention, that the weights were determined based on arguments and reasonable assumptions, as detailed herein, drawing on literature reports where experts’ opinions have already been taken into account.

A few assumptions made in this AHP application: (1) smugglers attack and seize the whole ship or (2) the oil that is being transferred. Further assumptions also made for the four criteria and discussed below:

1. *Security*: It is more secure for the smugglers to seize ships in: (a) territorial waters than in international waters and (b) international waters than in ports. Herein, we assume that it is much more dangerous for the pirates to attack in the ports, since the port authorities are based there. In addition, in territorial waters the ships move slower than in international waters and it is easier for the smugglers to board on it, thus minimizing the danger of engagement, and there is a lower level of security in comparison with international waters (Murphy, 2007).
2. *Time*: It is more time-consuming to seize ships in international waters, than in territorial waters and ports as the endpoints (e.g., warehouses etc.) that are needed to transfer the oil are on the land (Hastings, 2009).
3. *Topography*: Regarding challenges such as the wind, weather, underwater obstacles etc., it is better for smugglers to attack in the ports or territorial waters than in deep sea. Many incidents take place in or near narrow, congested channels that serve as chokepoints for world commerce, such as the Bab al-Mandab at the southern tip of the Red Sea, and the Phillips and Singapore Straits at the southern terminus of the Strait of Malacca in Southeast Asia. In such places, ships must slow down to navigate through shallow waters and underwater obstacles, and avoid other vessels, leaving them open to attack by land-based pirates (Murphy, 2009).
4. *Fuel consumption*: It is cheaper to attack ships near ports than in territorial or international waters because the end point is on land.

### Spatial, temporal, and spatiotemporal analyses

Spatial analysis, a field of science to shed light on patterns and underlying processes of different phenomena in terms of space, includes several tools. In this analysis, we implement kernel estimators and K-means clustering. Both tools use in, at their core, the measure of distance between piracy hits. Their distinct difference lies in using a grid point in the former tool compared to using the entire region of study in the case of K-means. Simply put, kernel estimators output information on the density of events per grid. K-means works supplementarily by clustering these events based on their proximity to the entire area.

Kernel estimators attempt to describe hidden spatial point patterns in terms of their probability density functions p(s) and intensity functions λ(s). The probability density function is defined as the probability of observing a point, or in our case, an event, at a specific location of a study region. In contrast, the intensity function λ(s) is the expected number of events per unit area at this location of the study region. Said that we follow Waller and Gotway (2004), explore the existence of spatial point patterns in our data set N of 2145 point observations, and employ the abovementioned estimates of probability density function p(s) and intensity function λ(s) as follows:

1 $$ \widehat{p( {s_{g} } )} = \frac{{\hat{\lambda }\left( {s_{g} } \right)}}{{\sum\nolimits_{j = 1}^{G} {\hat{\lambda }\left( {s_{j} } \right)} }} $$

2 $$ \hat{\lambda }\left( {s_{g} } \right) = \frac{C}{{A_{g} }}\sum\limits_{i - 1}^{n} {K\left( {{\raise0.7ex\hbox{${d_{ig} }$} \!\mathord{\left/ {\vphantom {{d_{ig} } {h_{g} }}}\right.\kern-0pt} \!\lower0.7ex\hbox{${h_{g} }$}}} \right)} \cdot y_{i} $$

where *K* (.) is the weight applied to the number of events *y*_*i*_ located at our data point *s*_*i*_ based on the quartic kernel function,* s*_*g*_ is the grid point derived from a grid we have generated to cover the area of interest, *d*_*id*_ is the Euclidean distance between our data point and the grid point, and *h*_*g*_ is the kernel’s smoothing factor, i.e., its radius. *A*_*g*_ is the area over which the kernel function is evaluated at grid point s_g_, and *C* is a proportionality constant.

Continuing spatial pattern exploration, we employ a K-means clustering of our events. We use the default Euclidean distance as a similarity measure, calculated in kilometers from each hostility's latitude and longitude coordinates. We partition our events into ten sets to minimize the within-cluster variance. The selection was based on the visualization of these events on the world map. It was observed that piracy incidents are mainly located in three areas: (1) on the west coast of Africa off Nigeria and Cameron (AREA I), (2) between the east coast of Africa and West India (AREA II), and (3) the broader region of Indonesia (AREA III). Considering some apparent congregating of events in the area east of Africa, a total number of ten clusters is a good selection for our analysis.

In this part an attempt was made to explore if pirates, e.g., from AREA_i,_ are affected by the activity of the pirates from AREA_j_, or in other words, if information concerning the attacks from one area is traveling to the other areas, triggering a sequence of attacks or events in the specific location. To examine this, we carefully select standard and time-varying Granger causality tests based on the type of our data and employ them in a pair-wise mode in the three-time series with events corresponding to the three areas described above. Granger causality is a statistical concept of causality that tests if the presence of a time series improves the prediction of another one and not the physical causality of a proven relationship between a cause and its effect. This explains why a two-way causality might be found, in contrast to the strict one-way physical causal relationship. We use Granger causality to study socioeconomic variables and phenomena where no fundamental or classical theory exists. We first run the usual descriptive statistics and the empirical (cumulative distribution function) CDFs of these three-time series of events and then employ a set of unit roots tests to assess the order of their integration, which is a prerequisite for the next step of our temporal analysis. For robustness, we use the tests of Dickey, Fuller with GLS detrending (DF-GLS), of Phillips-Perron (PP), of Kwiatkowski, Phillips, Schmidt, and Shin (KPSS), of Ng and Perron (NP), and of Hylleberg, Engle, Granger, and Yoo. For the tests DF, PP, NP, and HEGY, the null hypothesis is non-stationarity; for the KPSS, the null hypothesis is that of stationarity. Depending on the stationarity results of our time series, we apply, in pairs, either the classic Granger causality test (Granger, 1969 and 1988) or the Toda-Yamamoto test (1995) as follows.

3 $$ y_{t} = \hat{\beta } + \widehat{A_{1}} y_{t - 1} + \cdots + \widehat{A_{p}} y_{t - p} + \underbrace {{ \cdots + \widehat{A_{p}} y_{t - p - d} + }}_{only\,for\,T - Y\,tests} \widehat{\varepsilon_{t}} $$

where *y*_*t*_, *β* and *ε*_*t*_ are n-dimensional column vectors and ${A}_{k}$ is an *n* × *n matrix* of parameters for lag *k.* The error or stochastic disturbance term is introduced to account for any sources of uncertainty or variability that independent variables cannot explain. It surrogates all variables that are omitted in the model. In our case, given the pair-wise mode, n equals to two; thus, the former equation can be written in matrix notation, using the standard bivariate VAR(m), as follows:

4 $$ y_{t} = \Pi x_{t} + \varepsilon_{t} $$

*where* $$y_{t} = \left[ {y_{1t} y_{2t} } \right]^{\prime }$$

$$ \begin{aligned} & x_{t} + \left[ {1y_{t - 1}^{\prime } y_{t - 2}^{\prime } \cdots y_{t - k}^{\prime } } \right]^{\prime } \\ & \Pi_{{2X\left( {2m + 1} \right)}} = \left[ {\Phi_{0} \,\Phi_{1} \, \cdots \Phi_{m} } \right] \\ & \Phi_{0} = \left[ {\varphi_{0}^{(1)} \,\varphi_{0}^{(2)} } \right]^{\prime } \,\& \,\Phi_{k} = \left[ {\begin{array}{*{20}c} {\varphi_{1k}^{(1)} } & {\quad \varphi_{2k}^{(1)} } \\ {\varphi_{1k}^{(2)} } & {\quad \varphi_{2k}^{(2)} } \\ \end{array} } \right]\,for\,k = 1, \ldots ,m \\ \end{aligned} $$

The null hypothesis of no Granger causality from variable ${y}_{i} to {y}_{j}$ is then *H*_0_: *R*_*i*→*j*_·*vec*(*П*), where *R*_*i*→*j*_ is the coefficient restriction matrix and *vec*(*П*) is the row vectorization of *Π.* The model notation for Toda-Yamamoto is the same but form, which is replaced by $m^{\prime}$ = m + d, with d standing for the additional lags for the maximum order of integration.

Although these causality tests are attractive because they do not need guidance from economic theory or for the use of simple reduced-form VAR models, and in the case of the Toda-Yamamoto test, for its basis on standard asymptotic distribution regardless of the size of integration and cointegrating properties, still they face issues regarding structural stability (Psaradakis et al., 2005; Swanson, 1998). To overcome this, we apply time-varying Granger causality tests with three recursive testing algorithms, namely the forward expanding (FE) window, the rolling (RO) window, and the recursive evolving (RE) window (Phillips et al., 2015; Shi et al., 2018, 2020). If we consider a sample of T + 1 observations {*y*_0_, *y*_1_, …, *y*_*T*_}, a number w, satisfying 0 < w < 1, and *T*_*w*_ = *integer* (*T*·*w*) then the time-varying Wald statistic, ${WS}_{w1,w}$ is computed over the subsample $\left\{ {y_{{T_{w1} }} , \ldots ,y_{{T_{w} }} } \right\}$. In the FE algorithm, a series of Wald-test statistics ${WS}_{w1,w}$ are estimated, with w_1_ = 0 and$w  \in  [{w}_{min}, 1]$, in the RO algorithm, the statistic is being estimated in a rolling window of constant size, running all the samples, and in the RE algorithm, we obtain test statistics for any subsample having the size ${T}_{{w}_{min}}$ or larger in a repeated procedure, providing a common end point of all the subsamples. All the obtained values of the Wald-Test statistic are compared with the 90th and 95th percentiles of bootstrap statistics explained in the works of Shi et al. (2018, 2020).

An additional hypothesis tested in this paper is about if the pirates from an area have underlying relationships or are known with the other pirates of the same location or are there multiple different networks within an area that operate independently. The question is of high importance. If pirates belong to or use, more or less, the same networks and people, then it would be more efficient to control these areas first since capturing one pirate in this area could bring another to light with fewer resources, effort, and time. We accept that, if pirates belong or use the same networks, then we should have frequent hits that are close in time and space. To examine this hypothesis, we applied Tobler’s concept of spatial autocorrelation, which in Tobler’s words, says that “*Everything is related to everything else, but near things are more related than distant things*” (Tobler, 1970), and use the global index of spatial autocorrelation of Moran’s* I* (Boots & Tiefelsdorf, 2000)*.* Nevertheless. this index alone cannot give a straightforward answer to our research question. Hence, we include the time dimension${tn}_{i}$, which we call it “time-neighboring”, as the variable of interest in the Moran’s index I. This value is no other than the average time interval between the event_t-1_ and event_t,,_ and event_t_ and event_t+1,_:

5 $$ tn_{i,t} = \left. {\frac{{d(event_{t + 1} - event_{t - 1} )\left( {days} \right)}}{2}} \right|_{{event_{t} = event_{i,t} }} $$

events $(t+1) \& (t-1)$ are the immediate following and preceding events from event $(t)$, from all the ${i}^{th}$ events of the same area.

Low values suggest events close in dates, while the opposite is true for high values.

In general terms, there are four steps when computing a global index of spatial autocorrelation:

- Set the variable of interest to be examined for spatial correlation, in our case it is the *time-neighboring* variable ${tn}_{i}$,.
- Compute the similarity level, ${s}_{ij}$, between all possible location pairs $(i,j)$. Our pairs are in coordinates of longitude and latitude.
- Apply appropriate weights, by multiplying variables of similarity level ${s}_{ij}$, and of spatial proximity, ${w}_{ij}$.
- Sum up all the products of the former step and divide it by a constant that stands for proportionality.

In our case the Moran’s index becomes as follows (Moran, 1948):

6 $$ I = \frac{{\sum\nolimits_{i = 1}^{N} {\sum\nolimits_{j = 1}^{N} {w_{ij} \left( {tn_{i} - \overline{tn} } \right)\left( {tn_{j} - \overline{tn} } \right)} } }}{{\frac{1}{N}\sum\nolimits_{i = 1}^{N} {\left( {tn_{i} - \overline{tn} } \right)^{2} \sum\nolimits_{i = 1}^{N} {\sum\nolimits_{j = 1}^{N} {w_{ij} } } } }} $$

where the similarity variable ${s}_{ij}$ is the $\left( {tn_{i} - \overline{tn} } \right)\left( {tn_{j} - \overline{tn} } \right)$ and the proximity variable ${w}_{ij}$ is calculated, as follows:

7 $$ w_{ij} = \left\{ {\begin{array}{*{20}l} {0,} \hfill & {if\,d_{ij} \le lb\,\& \,d_{ij} \le ub} \hfill \\ {1/d_{ij,} } \hfill & {if\,lb \le d_{ij} \le ub} \hfill \\ \end{array} } \right. $$

where ${d}_{i,j}$ denotes the Euclidean distance, lb is the lower bound of the specified distance band, *ub* is the upper bound of the specified distance band.

Based on the steps described above and on the interpretation of Moran’s I index, we can make the following hypothesis:

- *Null Hypothesis*, H_o_: Time neighboring has no spatial pattern (spatial randomness)
- *Alternative Hypothesis*, H_a_ (with Z-score > 0): High and Low values of Time-neighboring are more spatially clustered than would be expected with spatial randomness.
- *Alternative Hypothesis*, H_a_ (with Z-score < 0): High and Low values of Time-neighboring are more spatially dispersed than would be expected with spatial randomness.

Our research question could be answered in positive, in the case of significant *p*-values with positive Z-score. In this case, among others, we could infer that there are events clustered with low values of time-neighboring, or in other words, we have events close enough in time and space that might be attributed to having common or even the same networks or persons.

## Results and discussion

### AHP findings

AHP method was applied by creating four tables, each for every abovementioned criterion to find the best alternative that satisfies the criteria (Saaty, 1987). For example, regarding the security criterion, smugglers will consider attacking near ports as the degree of security is high while the opposite is true, for territorial waters. Therefore, a higher score will be given to the area of the territorial waters than the ports, under the security criterion. In the same way, we repeat the procedure for the other three criteria (i.e., Time, Topography and Fuel Consumption). The results -indicatively- for security criterion are presented in Table 3.

According to Table 3, we obtain for the vector of relative weights: (Security, Topography, Time, Fuel Consumption) = (0.643, 0.056, 0.179, 0,122) with a CI = 0.0547 < 0.10, which is accepted according to the literature (Saaty, 1987).

Then, we repeat the same procedure for the principal eigenvector for the other alternatives, based on the rest of the criteria. We can realize that under security criterion, the smugglers will think probably to attack in territorial waters and not in international waters or near ports. As we mentioned above, there is enhanced security near the ports due to port authorities which is deterring smugglers to attack the ships. The overall weights occurring for the three alternatives strategies under the criterion “Security” are the following:

$$ \left( {{\text{Ports}}, \, \,{\text{Territorial}},\,{\text{ International}}} \right) \, = \, \left( {0.0{81}, \, 0.{731}, \, 0.{188}} \right). $$

Consecutively, the local derived scales for the three other criteria are the following:

$$ \left( {{\text{Ports}},\,{\text{ Territorial}},\,{\text{ International}}} \right) \, = \, \left( {0.{727}, \, 0.{2}00, \, 0.0{73}} \right). $$

$$ \left( {{\text{Ports}},\,{\text{ Territorial}},\,{\text{ International}}} \right) \, = \, \left( {0.{661}, \, 0.{272}, \, 0.0{67}} \right). $$

$$ \left( {{\text{Ports}}, \, \,{\text{Territorial}},\,{\text{ International}}} \right) \, = \, \left( {0.{692}, \, 0.{231}, \, 0.0{77}} \right). $$

Finally, based on the above information we multiply the two matrices below to find the final weight for each alternative according to the AHP analysis.

$$ \left[ {\begin{array}{*{20}c} {0.081 } \\ {0.731 } \\ {0.188 } \\ \end{array} \begin{array}{*{20}c} {\quad 0.727 } \\ {\quad 0.200 } \\ {\quad 0.073 } \\ \end{array} \begin{array}{*{20}c} {\quad 0.661 } \\ {\quad 0.272 } \\ {\quad 0.067} \\ \end{array} \begin{array}{*{20}c} {\quad 0.692} \\ { \quad 0.231} \\ { \quad 0.077} \\ \end{array} } \right] \times \left[ {\begin{array}{*{20}c} {0.643} \\ {0.056} \\ {0.179} \\ {0.122} \\ \end{array} } \right] = \left[ {\begin{array}{*{20}c} {0.295} \\ {0.558} \\ {0.147} \\ \end{array} } \right] $$

Based on the above calculations, the second alternative- the territorial waters-, is the most preferred strategy with the highest score of 0.558. Therefore, most of the times the smugglers prefer to attack in territorial waters, fewer times near ports, and rarely in international waters. This is in line with other studies, as there is no great effect of piracy in international waters, after analyzing real life events. Finally, it is essential to mention that our analysis with the use of AHP method is not limited to specific places or countries, but it takes a holistic perspective.

### Spatio-temporal data analysis

**Fig. 1:**
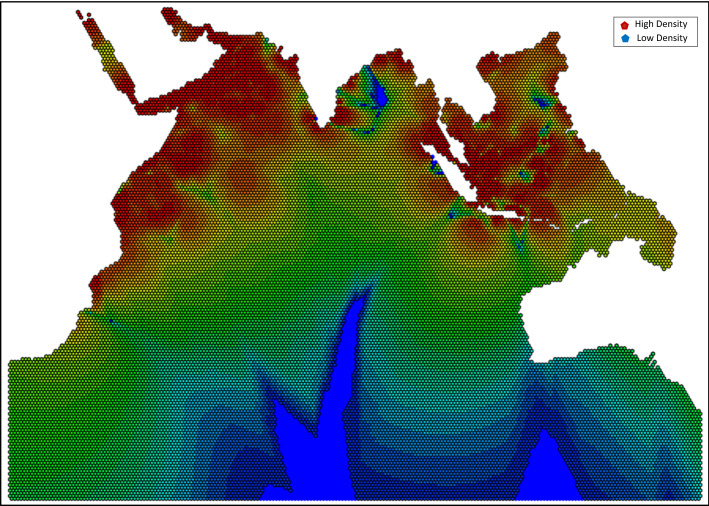
Kernel estimates of the Probability densities of attacks in areas II and III

**Fig. 2:**
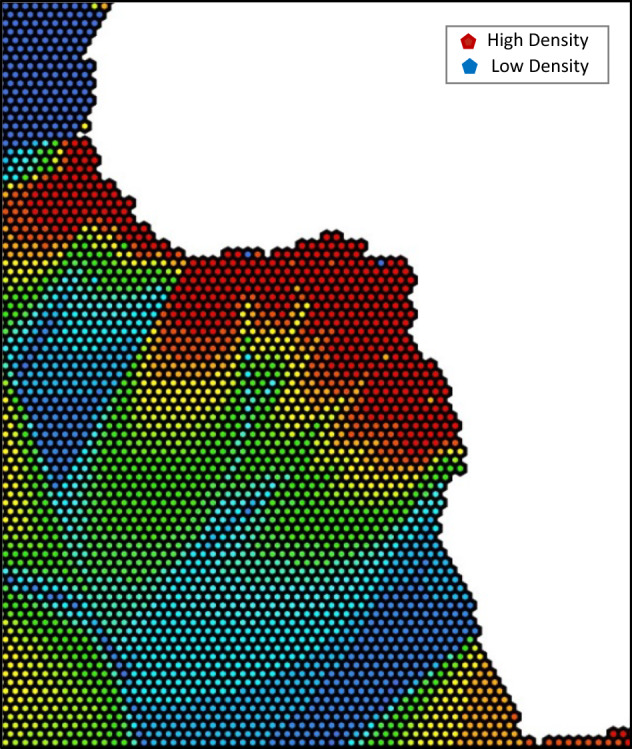
Kernel estimates of the Probability densities of attacks in area I

On spatial pattern recognition, Figs. 1 and 2 show the kernel estimates of the probability density of events for areas II and III and area I, respectively. The quartic kernel function has been used with the minimum (weighted) number of data points as the kernel bandwidth method.

**Fig. 3:**
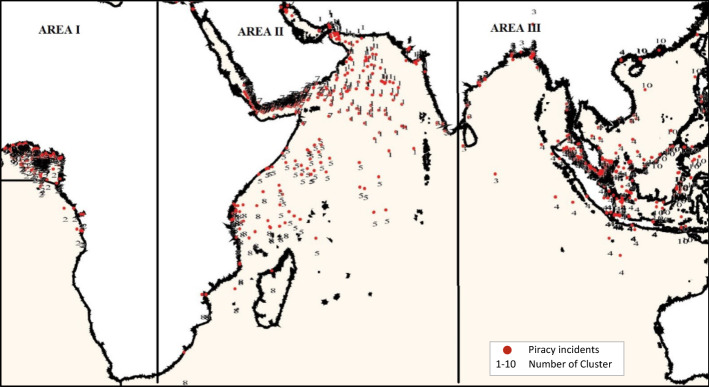
K-means clustering of the attacks in all areas

The mapped, in Figs. 1 and 2, probabilities of the events suggest that pirates prefer to hit close to the coastline, except for the Arabian sea (the only open sea). Also, pirates prefer the coasts of countries ranked low in surveillance or control from government forces, and in general, underdeveloped countries or face wars, riots etc. Next, in Fig. 3, we see the K-means clustering of the events. Area II has the most clusters, four out of ten, followed by area III with three clusters, and area I with two clusters. Cluster 6 is on the coasts of south and central America and does not belong to any of the three areas.

**Fig. 4:**
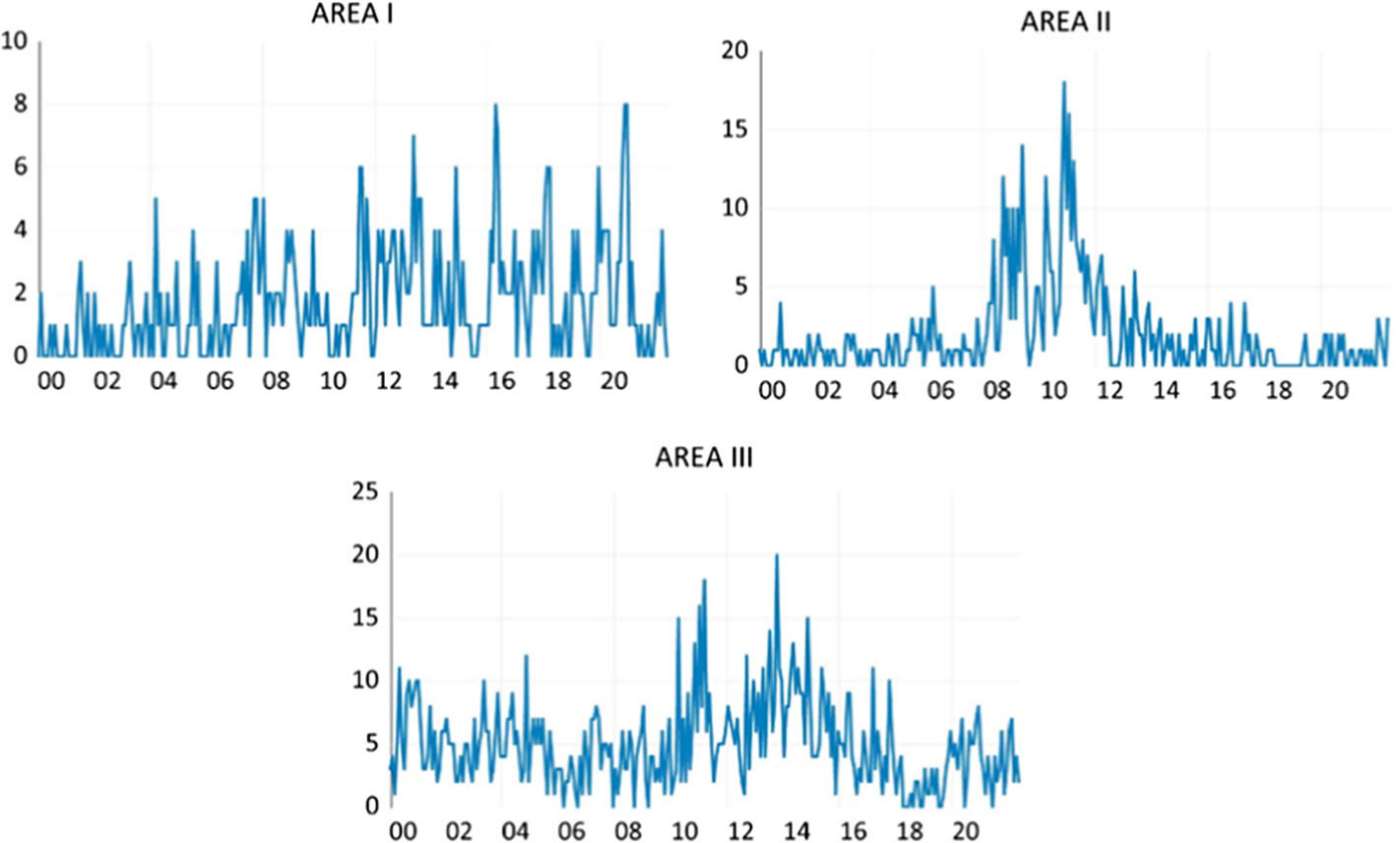
Monthly attacks against tankers in areas I, II and III for the period 2000–2020

**Fig. 5:**
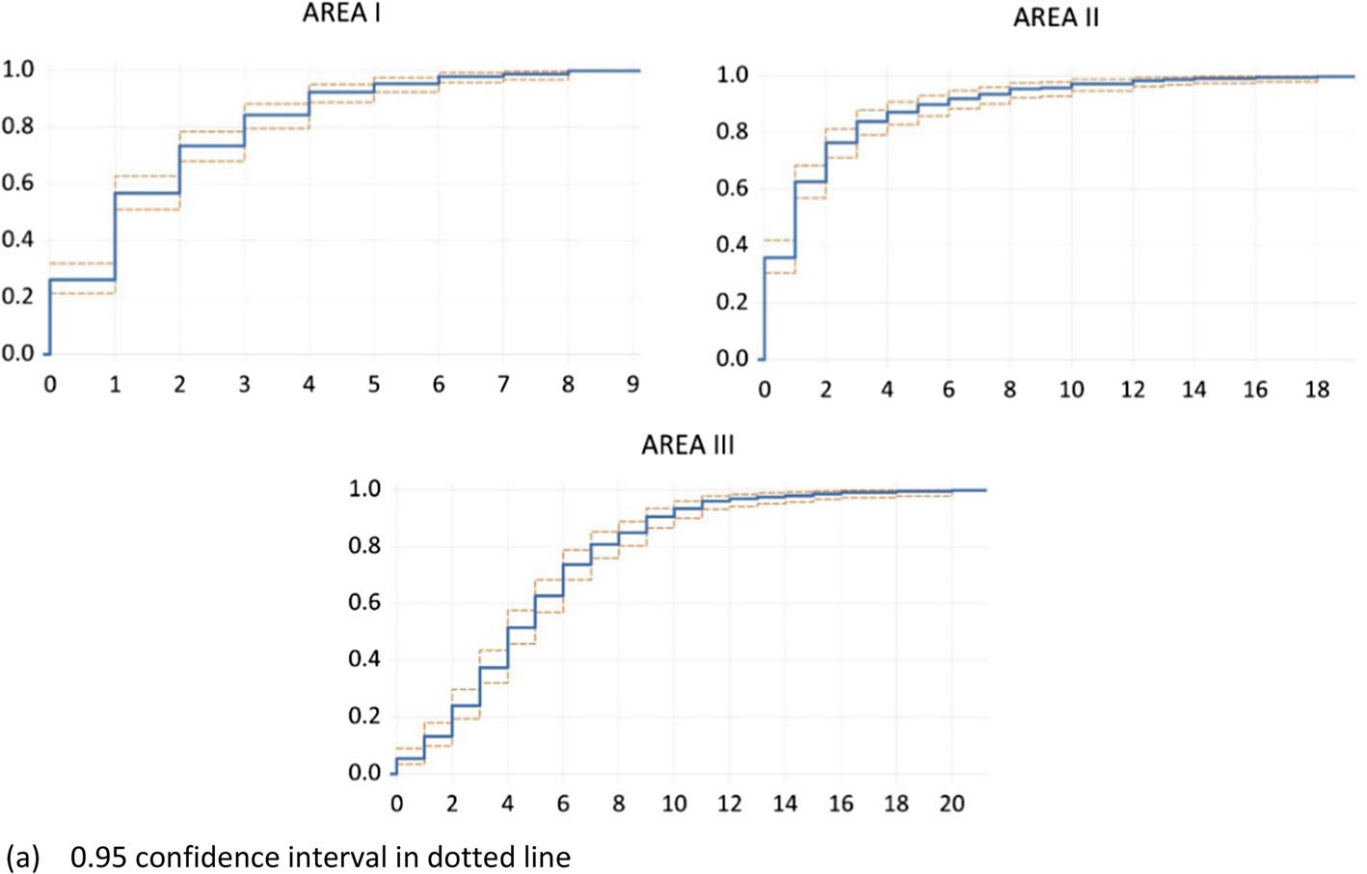
Empirical CDFs of the monthly attacks of areas I, II and III for the period 2000–2020

The monthly attacks on tankers, their statistics, and their empirical CDFs, are shown in Fig. 4, Table 1 and Fig. 5 respectively.

As shown in Fig. 4, area II has two spikes of incidents around 2008 and 2010. Two tips also appear in area III, around 2010 and 2013, while for area I, a slightly increasing trend is recorded from 2000 until 2020.

The three areas of study exhibit different similarities and dissimilarities, making a precise match among them not straightforward. As shown in Fig. 5, the cumulative probability of two attacks per month is 0.6 for areas I and II, while area III is at six attacks per month. On the contrary, the asymptotic limit of the unit of the three CDFs suggests similar behaviors only for areas II and III, with approximately 12 attacks per month. In contrast, this is reached at half monthly episodes in area I.

The unit root test results in Table 5 answer in the negative in stationarity, except for area I, where the KPSS test reject’s the null hypothesis of stationarity.

Given the stationarity results, we implement the standard Granger causality test for areas II and III and the augmented Toda-Yamamoto test for the pairs, including area I. As shown in Table 6, the causality results suggest causality running only from area III to area I, for a significance level of 10%. No other causal relationships exist.

**Fig. 6:**
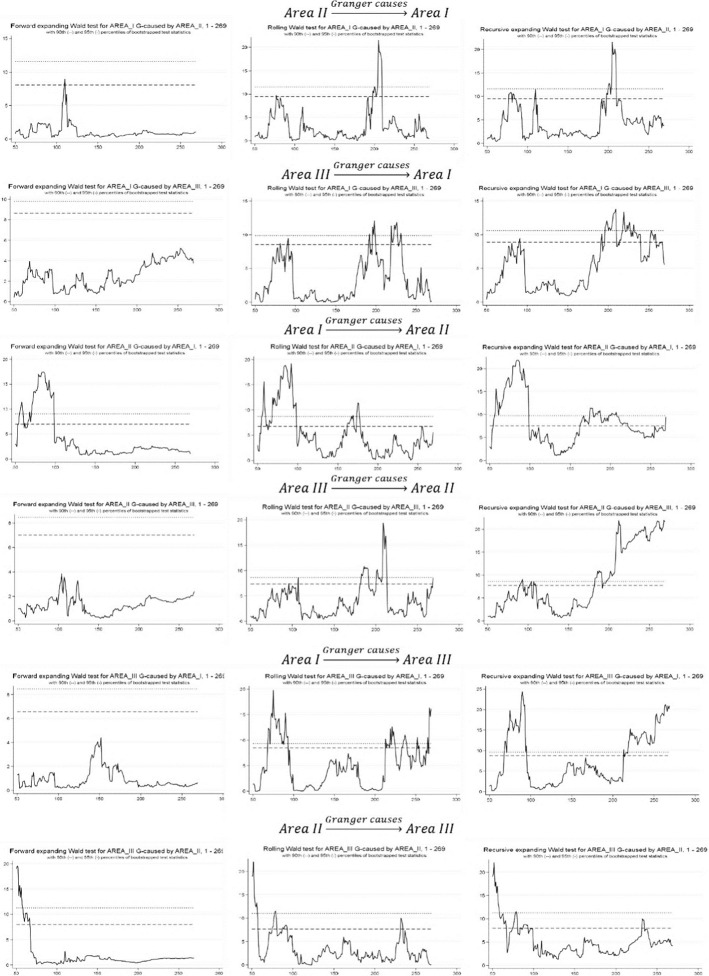
Series of Wald test statistics with FE, RO, and RE recursive algorithms

Although the Granger causality test is a helpful tool for detecting dependencies among time series, it may be fragile when different periods are examined just as with other structural stability issues. To address this issue, robust causality tests have been introduced, using time-varying methods and estimations (Rossi & Wang, 2019; Phillips et al., 2015; Shi et al., 2018, 2020). In Table 7 and Fig. 6 that follow, we show the time-varying Granger causality tests based on Shi et al. (2018, 2020).

**Table 7:** Statistics and Spearman’s correlation

	AREA I	AREA II	AREA III
Mean	1.73	1.96	4.94
Median	1.00	1.00	4.00
Maximum	8.00	18.0	20.00
Minimum	0.00	0.00	0.00
Std. Dev	1.73	2.91	3.38
Skewness	1.26	2.56	1.10
Kurtosis	4.42	10.57	4.92

*r*_*s*_^a^	AREA I	AREA II	AREA III
AREA I	1	–	–
AREA II	0.10	1	–
AREA III	0.14	0.21	1
Obs	269	269	269

^a^Spearman’s non-parametric correlation

The results in Table 7, for the entire sample, show that we fail to reject the null hypothesis of no causal relationship from area I to area III and vice versa and from area III to area II, when the FE recursive algorithm is used. A causal relationship exists in all other pairs in the three main areas (I, II and III), regardless of the algorithm used. Nevertheless, as shown in Fig. 6, these results show strong instability. In most cases, the produced series of Wald statistics exceed or fall behind the critical values depending on the time window and the recursive algorithm. Based on the 90th and 95th significance percentiles, shown in the dotted lines in Fig. 6, we see in detail in which time windows a causal relationship exists, that is when the test statistics of FE, RO and RE exceed these lines. Hence, if all algorithms and time windows are taken into account (see Fig. 6), causality exists only from area I to area II in the first 100 months (based on the three Wald statistics of FE, RO and RE), from area III to area II after the 200th month (based on RE algorithm), and from area I to area III, roughly, before the 100th month and after the 200th month (based on RO and RE algorithms).

Table 8 presents the results of the global index of spatial autocorrelation, Moran’s* I*, using the time-neighboring concept as the variable of interest (Tables 9, 10 and 11).

**Table 8:** Unit roots test results

Unit roots tests	DF-GLS test	PP-test	KPSS-test	NP -Test (MZ_a)_	HEGY seasonal test
AREA I	− 4.06***	− 6.89***	0.98***	− 29.47***	28.01***
AREA II	− 4.53***	− 5.82***	0.33	− 34.72***	36.24***
AREA III	− 3.68***	− 5.45***	0.24	− 21.20***	27.76***

In HEGY seasonal test, the periodicity has been set to 12 given our monthly data, t- statistic and critical values are for all seasonal frequencies jointly, results are identical with any frequency in separate

AREA I is I(1) based on the KPSS unit root test

^*^, ** and *** stand for 0.10, 0.05 and 0.01 level of significance

**Table 9:** Granger and Toda-Yamamoto causality test results

	$\mathop{\longrightarrow}\limits^{Granger\, Causes}$	${\chi }^{2}-test$	Prob
Toda-Yamamoto causality test^a^	$\mathrm{Area I}\to \mathrm{Area II}$	0.59	0.74
	$\mathrm{Area II}\to \mathrm{Area I}$	1.07	0.58
	$\mathrm{Area I}\to \mathrm{Area III}$	5.64	0.22
	$\mathrm{Area III}\to \mathrm{Area I}$	8.22*	0.08
Granger causality test	$\mathrm{Area II}\to \mathrm{Area III}$	1.02	0.36
	$\mathrm{Area III}\to \mathrm{Area II}$	1.67	0.19

^a^d_max_ = 1, Optimal lag length based on Information criteria, tests for serial correlation and stability condition are applied

*,** and *** stand for 0.10, 0.05 and 0.01 level of significance

**Table 10:** Maximum Wald-Statistics using the recursive algorithms of FE, RO and RE

Direction of causality	Max wald FE	Max wald RO	Max wald RE
Area I −> Area II	17.458	19.171	21.869
	− 6.989	− 6.729	− 7.521
	[9.070]	[8.693]	[9.749]
Area II −> Area I	8.977	21.525	21.525
	− 8.083	− 9.485	− 9.485
	[11.600]	[11.545]	[11.600]
Area I − > Area III	4.391	19.778	24.395
	− 6.558	− 8.501	− 8.77
	[8.466]	[9.346]	[9.607]
Area III −> Area I	5.217	12.061	13.783
	− 8.633	− 8.523	− 8.876
	[9.805]	[9.864]	[10.595]
Area II − > Area III	19.588	22.07	22.07
	− 8.015	− 7.71	− 8.015
	[11.256]	[11.000]	[11.256]
Area III −> Area II	3.84	19.382	21.956
	− 7.017	− 7.35	− 7.733
	[8.458]	[8.620]	[8.620]

Area I pairs use the time varying LA-VAR model of Toda- Yamamoto test

FE, RO, and RE stand for forward, rolling and recursive respectively

The critical values based on Chi- Square distribution at the 90th and 95th percentiles are in parentheses and brackets, respectively

**Table 11:** Moran’s I global index results

Area	*I* − stat	E (*I*)	St. dev(*I*)	Z-score	*P*-value
I	0.04***	0.002	0.01	6.61	0.00
II	0.10***	0.002	0.01	7.46	0.00
III	0.12***	− 0.001	0.01	27.11	0.00

Variable of interest is, for all areas, time-neighboring

A binary weights matrix has been created with threshold distance for neighbors the unit, 1-tail test has been used

*, ** and *** stand for 0.10, 0.05 and 0.01 level of significance

All areas have strong positive values of Z-scores and reject the null hypothesis of spatial randomness. In other words, it might be argued that in each of the three areas, pirates use the same or related networks or persons for their attacks since there are clusters where attacks happened to close to each other in space and time terms.

## Conclusions

Maritime piracy is a great concern as it affects not only the vessel and cargo but also leads to delays of ships and other socioeconomic impacts. Oil tanker companies try to reroute their ships or invest in security e.g. armed guards, water cannons. Despite the urgency of mitigation practices and the need to handle the piracy incidents systematically, only a few studies have been conducted that discuss the regularities in patterns of attacks and entail a spatio-temporal analysis to support decision-making towards piracy and interventions.

In this two-level analysis, AHP method applied first, to explore the causing factors and the most highly vulnerable areas of piracy. Various factors in the past have been identified as enablers of these incidents such as increased volume and value of a cargo, technological advances, and political instability (Min, 2011). Here, the focus was on the following criteria: zone, security, time, distance. It is found that most of the times smugglers prefer to attack ships in territorial waters rather in international waters. Thus, effective regional strategies and policies should be implemented to add more resources (e.g., sufficient resources for responding to emergencies, navy patrol protection) in the port and territorial areas.

In the second level, the spatiotemporal analysis suggests that there are specific clusters, namely on the west coast of Africa of Nigeria and Cameron (Area I), between the east coast of Africa and West India (Area II), and the broader region of Indonesia (Area III), where most attacks occur. Based on these areas it can be observed that pirates often prefer attacks close to the coastlines of underdeveloped countries or have no robust democracies. This is in line with the findings from the study of Desai (2021) who conducted a geographically disaggregated analysis. The challenges for combating maritime piracy derive from the lack of robust legal and institutional mechanism mostly at the domestic level (Ahmad, 2020). Local governments and multinational oil companies need to support and offer economic opportunities for coastal communities there.

Based on Fig. 4, we can see a that there is an increase of incidents of piracy and armed robbery against ships in Area II and Area III during 2008–2012 and 2010–2016 respectively. According to Oyewole (2016), in some of these areas there were more dedicated efforts that brought good governance and transparency and support these regions thus minimized the incidents. Therefore, it is really important each of these areas to implement their own national maritime security policies and strategies. These strategies and policies should be linked with financial, social, and political support that will strengthen the relevant institutions and enforcement mechanisms. Unfortunately, there would be some difficulties to implement them in some areas e.g., in Somalia due to low governance indicators (Alsawalqa & Venter, 2022). There is no pattern of the incidents in Area I but we can see that is on the rise. Apart from corruption, unemployment, the topography of this areas enables pirates to conceal ships, boats and stolen commodities in Nigeria’s coast i.e., inlets, rivers, and mangroves (Mishra, 2019).

Also, it is found that pirates in area II are influenced by the activity and the information coming from the pirates of areas I and III, suggesting a priority to control these two areas first. In addition, in all areas, the hypothesis of shared networks and persons from pirates has been verified, encouraging authorities to derive as much information as possible from pirates that have been arrested. Thus, these affected areas need to share information about these incidents on their coastlines and their neighbors.

This is in line with work from Phayal et al. (2022) that the likelihood of piracy in territorial waters is low when there is more security cooperation among bordering states. Thus, there is a need for stronger collaboration from policy perspective among EU, Asian, and African states.

The contributions of this study are three-fold. First, a two-step approach namely AHP method and spatiotemporal analysis applied to assess maritime piracy risks, until now there are only a few studies that investigated the causes of maritime piracy in a quantitative way. Second, this study took a holistic approach for identifying global hotspots, and third previous studies did not explore in detail the determinants of their presence. Last but not least, some interesting findings revealed that can help various stakeholders to apply more efficient and effective anti-piracy strategies. Even though this study explored and answered three important questions regarding causing factors, and hotspots of maritime piracy, future work may contain further analysis of policies that need to be developed and how to handle piracy in specific geographical areas, thus how interventions against maritime piracy may differ according to regions. Moreover, future studies could evaluate the costs of maritime security of conservation initiatives.

## Data Availability

The analyzed data during the current study are available in the National Geospatial-Intelligence Agency repository.
